# Survival status and predictors of mortality among children with severe acute malnutrition admitted to general hospitals of Tigray, North Ethiopia: a retrospective cohort study

**DOI:** 10.1186/s13104-018-3937-x

**Published:** 2018-11-26

**Authors:** Gebremicael Guesh, Getu Degu, Mebrahtu Abay, Berhe Beyene, Ermyas Brhane, Kalayu Brhane

**Affiliations:** 1 JSI, P.O. Box: 13898, Addis Ababa, Ethiopia; 2 Department of Epidemiology and Biostatistics, Institute of Public Health, University of Gondar, P.O. Box: 196, Gondar, Ethiopia; 3 School of Public Health, College of Health Science, Aksum University, P.O. Box: 298, Aksum, Ethiopia; 4 Department Public Health, College of Health Science, Debre Berhan University, P.O. Box: 445, Debre Berhan, Ethiopia

**Keywords:** Survival status, Severe acute malnutrition, Mortality, Tigray region

## Abstract

**Objective:**

Despite the presence standard protocol for management of severe acute malnutrition case-fatality rates in African hospitals remain unacceptably high. The case in Ethiopia is not different from others. Therefore, this study was aimed to assess survival status and predictors of mortality among children with severe acute malnutrition admitted to stabilization centers of general hospitals in Tigray region, northern Ethiopia. A 24 months retrospective longitudinal study was conducted among 569 randomly selected medical records of children admitted to stabilizing centers. Both bi-variable and multivariable Cox regression analysis was conducted to identify predictors of mortality. Association was summarized using AHR, and statistical significances were declared at 95% CI and P-value < 0.05.

**Results:**

During follow up, 456 [82%] of children had got cured, 37 [6.65%] were absconded and 21 [3.8%] were died. The overall mean survival time was 41.93 [95% CI 40.17–43.68] days. Impaired conscious level [AHR = 6.69, 95% CI 2.43–19.93], development of comorbidity after admission [AHR 12.71, 95% CI 2.79–57.94] and being urban in residence [AHR = 2.73, 95% CI 1.12–6.64] were predictors of mortality. Therefore, interventions to reduce further mortality should focus in children having impaired consciousness level and who developed comorbidity after admission.

**Electronic supplementary material:**

The online version of this article (10.1186/s13104-018-3937-x) contains supplementary material, which is available to authorized users.

## Introduction

Malnutrition refers to a pathological state in which children and adults are suffering from deficiency or excess of one or more nutrients leading to the point where the body can no longer perform proper body functions [1]. In developing countries, the deficiency states are common nutritional problems [2].

Sever acute malnutrition (wasting and/or nutritional edema) is defined globally as a very low weight for length/height (WFL/WFH) below – 3 zscores of the median WHO growth standards, or less than 70% of the median National Center for Health Statistics standard or the presence of nutritional edema. In children aged 6–59 months, mid-upper arm circumference (MUAC) less than 11.5 cm is also indicative of severe acute malnutrition [3].

Sever-acute malnutrition (SAM) remains a major killer of children under 5 years of age [4]. Children with severe acute malnutrition are nine times more likely to die than well-nourished children [5]. In 2016, 17 million children under 5 were affected by severe acute malnutrition globally, in which more than three-fourth of them are from South East Asia and sub-Saharan Africa with nearly half of under-five mortality attributable to malnutrition [6].

Despite the presence standard protocol for the management of SAM, case-fatality rates in African hospitals for SAM remain unacceptably high, especially in children complicated by HIV, invasive bacterial disease or underlying medical complications [7].

As one of the developing countries, the case in Ethiopia is not different from the others, more than half of deaths in young children are attributable to malnutrition [8]. SAM occurs in 11% of children [9], and is the reason for 19.3% of pediatric hospital admissions [10]. Unfortunately, more than one-fourth of deaths are occurring during hospital admission [4]. In different studies, the mortality rate due to SAM ranges from 6 to 28.67% [11, 12]. Studies suggest that the possible causes for high mortality rate could be alate presentation of cases and anemia [12–14], comorbidities like malaria and faulty in management [12], age less than 24 months, impaired consciousness level and hypothermia [14].

Despite the majority of Ethiopian children with severe acute malnutrition come to hospital or health center to be treated at therapeutic feeding center they die in the stabilizing centers (SCs). Death of many children in the presence of functional stabilizing centers following standard treatment protocols is not acceptable. Besides to this the major determining factors for poor treatment outcomes are not well understood particularly in the general hospitals of Tigray region. Therefore, the aim of this study is to assess survival status and predictors of mortality among children with SAM admitted to stabilization centers of general hospitals in Tigray region, northern Ethiopia.

## Main text

### Methods

#### Study design and setting

A facility-based retrospective longitudinal study was conducted from July to September 2016 in general hospitals of Tigray region, one of the regional states in the federal democratic republic of Ethiopia. The region is found in the northern part of the country 783 kms far from Addis Ababa, the capital city of Ethiopia. According to the 2016 annual Regional Health report the common causes of mortality among under-five children were malnutrition, pneumonia, diarrhea, and malaria [15].

#### Study population

All severely malnourished under-five children admitted to SCs of General Hospitals in Tigray from January 01, 2013 to December 30, 2015, were the source population.

#### Sample size and sampling technique

The sample size was calculated based on double population proportion formula by using Epi Info version 7 considering the following assumptions: 95% CI 80% power ratio of unexposed to exposed 2 outcome in exposed = 15.88% outcome in unexposed 7.85% and risk ratio of 2, and was found to be 569 [14]. The sample was allocated proportionally for the randomly selected hospitals, and simple random sampling technique was used to select individual records from registers. Children with incomplete records were excluded.

#### Study variables and their measurement

The event in this study was death. The time variable was time from admission to the occurrence of death coded as one otherwise zero and the independent variables were socio-demographic characteristics like age, residence and sex, co-morbidities and clinical presentation like type of malnutrition, presence of diarrhea, vomiting, dehydration, skin lesions, anemia, pneumonia, level of consciousness, conjuctival color, presence of shock and development of medical complication after admission, and routine medications. All the co-morbidities and medical complications were defined according to the national SAM management protocol [16].

#### Data processing and analysis methods

The collected data were checked for completeness and consistency and then, entered into Epi Info7 after providing a unique code for each questionnaire. The entered data were exported to STATA version 11 for analysis. Life table was constructed to estimate the probabilities of death over time. Kaplan–Meier survival curve together with log-rank test was fitted to test for the presence of a difference in the occurrence of death among the categorical variables.

Bi-variable Cox regression analysis was conducted to assess the effect of each independent variable on the outcome variable. Variables with P-value < 0.25 in the Bi-variable Cox regression analysis were included in the multivariable Cox regression analysis to identify the independent predictors of mortality. Proportionality of Hazard assumption was tested by global test based on Schoenfeld residuals. Association was summarized by using AHR, and statistical significances were declared at 95% CI and P-value < 0.05.

### Results

#### Socio-demographic characteristics and type of malnutrition

Out of the total 569 randomly selected records of SAM, data with necessary information was extracted from 556 (97.77%) of the records, and the remaining 13 (2.23%), the record (patient card) was not found.

More than half, 301 (54.1%) of the children enrolled in the study were male. Three-fourth, 416 (74.8%) of the study participants were from rural areas. The majority, 479 (86.2%) were aged < 2 years with mean age of 16.51 [± SD 11.084] months.

Nearly three-fourth, 392 (70.5%) of children enrolled in the study had marasmus, 126 (22.7%) and 38 (6.8%) had kwashiorkor and marasmus–kwashiorkor respectively (Table 1).

**Table 1:** Socio-demographic, nutritional and clinical characteristics of children with SAM admitted to SCs of general hospitals of Tigray, Ethiopia, from 2013 to 2015

Variable	Category	Survival status		Total N (%)
		Dead number (%)	Censored number (%)	
Age in months	0–23 months	17 (3.55)	462 (96.45)	479 (86.2)
	24 months and above	4 (5.2)	73 (94.8)	77 (13.8)
Sex	Male	11 (3.65)	290 (96.35)	301 (54.13)
	Female	10 (3.92)	245 (96.08)	255 (45.87)
Residence	Rural	11 (2.64)	405 (97.36)	416 (74.82)
	Urban	10 (7.14)	130 (92.86)	140 (25.18)
Type of SAM	Marasmus	12 (3.06)	380 (96.94)	392 (70.5)
	Kwashiorkor	7 (5.56)	119 (94.44)	126 (22.7)
	Marasmic kwashiorkor	2 (5.26)	36 (94.74)	38 (6.8)
Diarrhea	Yes	12 (4.9)	233 (95.1)	245 (44.1)
	No	9 (2.9)	302 (97.1)	311 (55.9)
Vomiting	Yes	7 (3.8)	175 (96.2)	182 (32.7)
	No	14 (3.7)	360 (96.3)	374 (67.3)
Color of conjunctiva	Pink	10 (2.2)	441 (97.8)	451 (81.1)
	Pale	11 (10.48)	94 (89.52)	105 (18.9)
Level of consciousness	Conscious	13 (2.5)	500 (97.5)	513 (92.3)
	Impaired	8 (18.6)	35 (81.4)	43 (7.7)
Edema	Yes	8 (5.5)	137 (94.5)	145 (26.1)
	No	13 (3.2)	398 (96.8)	411 (73.9)
Palmar pallor	Yes	5 (6.9)	67 (93.1)	72 (12.9)
	No	16 (3.3)	468 (96.7)	484 (87.1)
Skin lesions	Yes	4 (9.5)	38 (90.5)	42 (7.6)
	No	17 (3.3)	497 (96.7)	514 (92.4)
Dehydration	Yes	7 (5.3)	125 (94.7)	132 (23.7)
	No	14 (3.3)	410 (96.7)	424 (76.3)
Patient in shock	Yes	3 (21.4)	11 (78.6)	14 (2.5)
	No	18 (3.4)	524 (96.6)	542 (97.5)
Pneumonia	Yes	8 (5.7)	132 (94.3)	140 (25.2)
	No	13 (3.1)	403 (96.9)	416 (74.8)
Anemia	Yes	3 (6.7)	42 (93.3)	45 (8.1)
	No	18 (3.5)	493	511 (96.5)
CHF	Yes	1 (7.7)	12 (92.3)	13 (2.3)
	No	20 (3.7)	523 (96.3)	543 (97.7)

### Clinical conditions and co-morbidities

About two-thirds, 367 (66%) of the participants had co-morbidity/complications at admission, of these 44.1% and 25.2% of the patients had diarrhea and pneumonia respectively. dehydration was reported in 132 (23.74%) of the total sampled children of which 36 (27.7%) were severely dehydrated (Table 1).

### Survival status and treatment outcome

#### Survival status

A total of 566 children were followed for different periods: a minimum of 1 day and maximum 45 days with a median follow up period of 10 days. The study participants were assessed retrospectively for 6671 persons-day. The overall incidence density of death was 0.0032 deaths per 1000 persons-days. The cumulative survival probability at the end of 1st, 7th, 14th, 21th and 28th days were 99.5%, 98%, 96.4%, 92.7% and 89.1%, respectively. The overall mean survival time was 41.93 (95% CI 40.17–43.68) days.

#### Treatment outcome

Throughout the follow up period 456 [82% (95% CI 78.6–85.3)] of children had got cured and discharged, 37 (6.65%) were absconded, 25 (4.5%) had got medical referral, 21 [3.8% (95% CI 2.2–5.6)] were died, 8 (1.44%) were transferred out and 9 (1.6%) were right censored. The average length of hospital stay was 12 (95% CI 11.42–12.59) days.

#### Factors associated with survival status of children with SAM admitted to SCs

In the bi-variable Cox regression, seven variables (namely: residence, presence of skin lesions, patient in shock at admission, the presence of comorbidity at admission, conjunctiva color, level of consciousness and development of comorbidity after admission) were found to be predictors of mortality.

In the multivariable Cox regression analysis, only three variables were found to be significant predictors of mortality. These were residence (being urban resident AHR = 2.73), impaired level of consciousness (AHR = 6.96) and development of comorbidity after admission (AHR = 12.71).

**Fig. 1:**
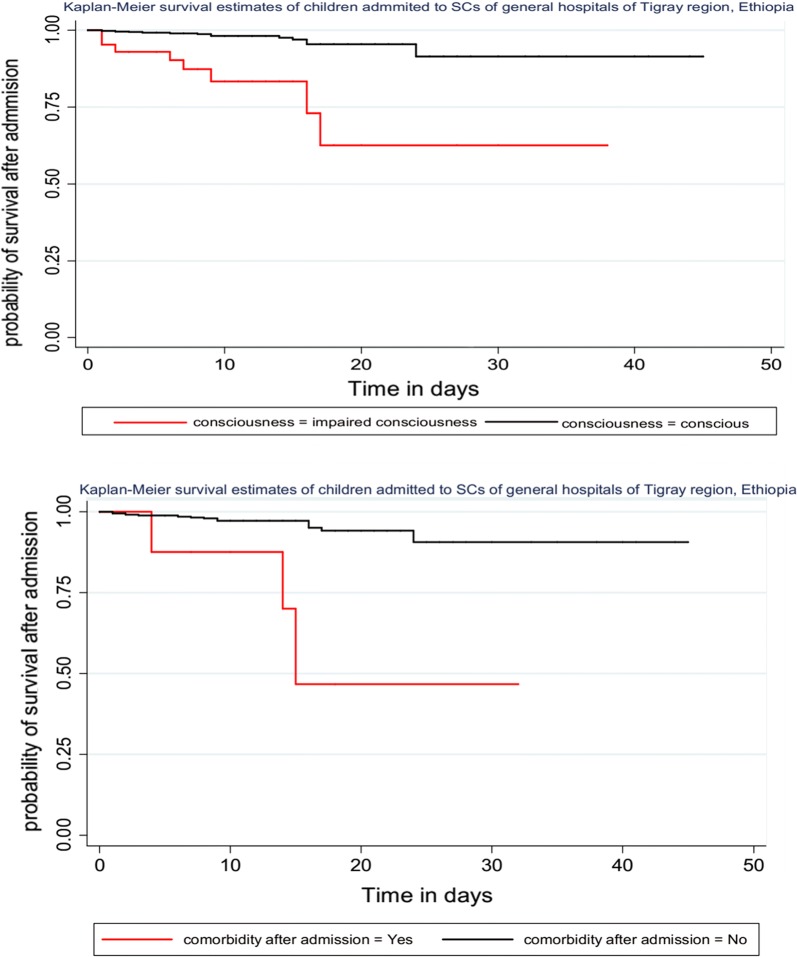
Kaplan–Meier survival plot of factors associated with survival status of children with severe acute malnutrition admitted to general hospitals of Tigray, North Ethiopia

Specifically, the risk of mortality among children with SAM admitted to SCs from the urban areas were 2.73 times higher as compared to rural residents (AHR = 2.73, 95% CI 1.12–6.64). the hazard of mortality among children with SAM admitted to SCs having impaired consciousness was 6.96 times higher as compared to conscious children (AHR = 6.69, 95% CI 2.43–19.93) Children who developed comorbidity after admission was 12.71 times at risk of mortality as compared to children who did not develop comorbidity after admission (AHR = 12.71, 95% CI 2.79–57.94) (Table 2 and Fig. 1).

**Table 2:** Bi-variable and multivariable analysis of predictors of mortality among children with SAM admitted to SCs of general hospitals of Tigray, Ethiopia, from 2013 to 2015

Variable	Survival status		CHR (95% CI)	AHR (95% CI)
	Death number (%)	Censored number (%)		
Residence				
Urban	10 (7.14)	130 (92.86)	3.56 (1.51–8.49)	2.73 (1.12–6.64)*
Rural	11 (2.64)	405 (97.36)	1	1
Level of consciousness				
Impaired	8 (18.6)	35 (81.4)	8.41 (3.47–20.34)	6.69 (2.43–19.93)**
Conscious	13 (2.5)	500 (97.5)	1	1
Comorbidity after admission				
Yes	3 (37.5)	5 (62.5)	9.76 (2.86–33.33)	12.71 (2.7957.94)**
No	18 (3.3)	530 (96.7)	1	1
Patient in shock				
Yes	3 (21.4)	11 (78.6)	6.01 (1.77–20.45)	2 (0.51–7.84)
No	18 (3.4)	524 (96.6)	1	1
Comorbidity at admission				
Present	17 (4.6)	350 (95.4)	2.38 (0.8–7.1)	1.08 (0.33–3.55)
Absent	4 (2.1)	189 (97.9)	1	1
Skin lesion				
Yes	4 (9.5)	38 (90.5)	2.29 (0.76–6.94)	0.79 (0.23–2.76)
No	17 (3.3)	497 (96.7)		
Color of conjunctiva			1	1
Pale	11 (10.48)	94 (89.52)	4.53 (1.92–10.68)	2.07 (0.78–5.5)
Pink	10 (2.2)	441 (97.8)	1	1

*COR* crude odds ratio, *AOR* adjusted odds ratio, *CI* confidence interval

* P < 0.05, ** P ≤ 0.001

### Discussion

This study found that the overall mean survival time was 41.93 (95% CI 40.17–43.68) days. Impaired conscious level, development of comorbidity after admission and being urban in residence were the independent predictors of mortality.

This study revealed that 21 (3.8%) children had died during the follow-up period. This result is in line with the study conducted in Southern Ethiopia, and Felegehiwot hospital, Ethiopia where the death was 3.6% and 2.76%, respectively [17, 18]. This result is also in agreement with the minimum SPHERE standard and national management protocol for severe acute malnutrition managed at stabilization centers (< 10%) [19].

But the death rate in this study is found to be slightly higher form the result reported from Ghana in which the death rate was zero percent [20]. The slight difference can be explained by the present study was conducted among children admitted to SCs whereas the previous study was conducted among children in outpatient care.

In contradiction to the above the death rate in this study is lower as compared to studies conducted in Mekelle hospital, Ethiopia (12.8%), Sekota hospital, Ethiopia (28.67%) and Zewditu hospital, Ethiopia (21.3%), respectively [12, 21, 22]. The difference can be due to differences in management team and supplies, patient load, patient clinical profile and a difference in following management protocol.

The average length of stay in the SCs in this study was 12 day which is consistent with the result reported from the study in Gedeo zone, southern Ethiopia, in which the average length of stay was 14 days [13]. However, this is much lower than from the international standard (SPHERE) set for the management of SAM which is < 30 days [19]. In contradiction to the above, this result was found to be longer as compared to other reported studies from Zambia (3 days) [23].

The difference can be explained by the difference in clinical profiles of children when children developed chronic commodities like TB and HIV/ADIS they may spend more time in hospitals [16].

The hazard rate of death among children with impaired consciousness level (coma or lethargic) at admission was 6.69 times higher as compared with children who were conscious at admission. A similar finding was reported from the study conducted in Jimma University specialized hospital [14]. This can be explained by impaired consciousness level may complicate the management of children with SAM.

Children who developed comorbidities after admission were 12.71 times at risk of earlier death as compared with children who did not develop comorbidities after admission. This could be due to increase nutrient loss and nutrient requirement in the face of decreased nutrient absorption and utilization. Besides to this the development of comorbidities after admission may complicate the management children with SAM.

The hazard rate of death among children who were from urban areas was 2.73 times higher as compared with children from rural areas. No similar finding was reported from the previous studies. This can be explained by the majority (74.8%) of the children in this study was from the rural areas and the total number of death observed is small as compared to the number of children from the rural areas. The finding of this research may provide necessary information on areas of improvement; however, further research is needed to give policy-level recommendation.

### Conclusion

The overall mean survival time was 41.93 (95% CI 40.17–43.68) days. Treatment outcomes were in an acceptable level of SPHERE standard, national management protocol and most reports in the literature. Impaired consciousness level, development of comorbidity after admission and being urban in residence were the independent predictors of mortality. Therefore, sound diagnosis and management of cases in SCs according to the national protocol is in need with special emphasis to children with impaired consciousness level and who developed comorbidities after admission.

### Limitation of the study

Due to retrospective nature of the study, some variables might be incomplete, while others were not recorded at all. Moreover, since majority of observations were censored, there may be potential bias due to excluded records and unknown status of absconders (Additional file 1).

## Additional file

**Additional file 1.** Survival data with minimal data.

## Abbreviations

ADIS: acquired immune deficiency syndrome
AHR: adjusted hazard ratio
CHR: crude hazard ratio
CMAM: community based management of acute malnutrition
EDHS: Ethiopia Demographic Health Survey
HIV: human immunodeficiency virus
MUAC: mid upper arm circumference
NCHS: National Center for Health Statistics
ReSoMaL: recommended solution for malnutrition
SAM: severe acute malnutrition
SCs: stabilizing centers
SD: standard deviation
TB: tuberculosis
TFU: therapeutic feeding unit
VitA: vitamin A
WHO: World Health Organization
WHA: weight for age-z score
WHZ: weight for height-Z score
